# Contributions of CO_2_, O_2_, and H_2_O to the Oxidative Stability of Solid Amine Direct Air Capture Sorbents at Intermediate Temperature

**DOI:** 10.1021/acsami.3c08140

**Published:** 2023-09-29

**Authors:** Yoseph A. Guta, Juliana Carneiro, Sichi Li, Giada Innocenti, Simon H. Pang, Miles A. Sakwa-Novak, Carsten Sievers, Christopher W. Jones

**Affiliations:** † School of Chemical & Biomolecular Engineering, Georgia Institute of Technology, 311 Ferst Dr., Atlanta, Georgia 30332, United States; ‡ Lawrence Livermore National Laboratory, 7000 East Avenue, Livermore, California 94550, United States; § Global Thermostat, 10275 E106th Avenue, Brighton, Colorado 80601, United States

**Keywords:** DAC, carbon capture, degradation, poly(ethylenimine), oxidation, radical

## Abstract

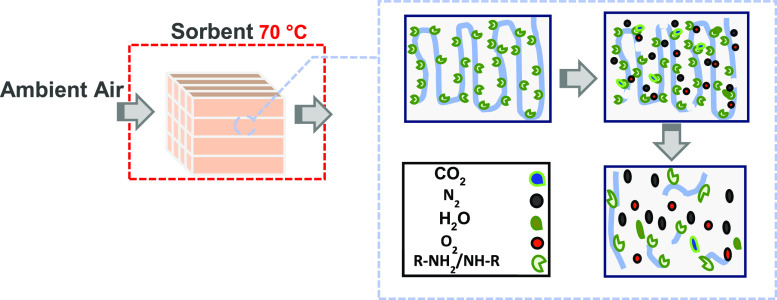

Aminopolymer-based sorbents are preferred materials for extraction of CO_2_ from ambient air [direct air capture (DAC) of CO_2_] owing to their high CO_2_ adsorption capacity and selectivity at ultra-dilute conditions. While those adsorptive properties are important, the stability of a sorbent is a key element in developing high-performing, cost-effective, and long-lasting sorbents that can be deployed at scale. Along with process upsets, environmental components such as CO_2_, O_2_, and H_2_O may contribute to long-term sorbent instability. As such, unraveling the complex effects of such atmospheric components on the sorbent lifetime as they appear in the environment is a critical step to understanding sorbent deactivation mechanisms and designing more effective sorbents and processes. Here, a poly(ethylenimine) (PEI)/Al_2_O_3_ sorbent is assessed over continuous and cyclic dry and humid conditions to determine the effect of the copresence of CO_2_ and O_2_ on stability at an intermediate temperature of 70 °C. Thermogravimetric and elemental analyses in combination with in situ horizontal attenuated total reflection infrared (HATR-IR) spectroscopy are performed to measure the extent of deactivation, elemental content, and molecular level changes in the sorbent due to deactivation. The thermal/thermogravimetric analysis results reveal that incorporating CO_2_ with O_2_ accelerates sorbent deactivation using these sorbents in dry and humid conditions compared to that using CO_2_-free air in similar conditions. The in situ HATR-IR spectroscopy results of PEI/Al_2_O_3_ sorbent deactivation under a CO_2_-air environment show the formation of primary amine species in higher quantity (compared to that in conditions without O_2_ or CO_2_), which arises due to the C–N bond cleavage at secondary amines due to oxidative degradation. We hypothesize that the formation of bound CO_2_ species such as carbamic acids catalyzes C–N cleavage reactions in the oxidative degradation pathway by shuttling protons, resulting in a low activation energy barrier for degradation, as probed by metadynamics simulations. In the cyclic experiment after 30 cycles, results show a gradual loss in stability (dry: 29%, humid: 52%) under CO_2_-containing air (0.04% CO_2_/21% O_2_ balance N_2_). However, the loss in capacity during cyclic studies is significantly less than that during continuous deactivation, as expected.

## Introduction

Global greenhouse gas (GHG) emissions from various economic sectors have been growing rapidly for several decades.^1^ GHGs present in the atmosphere absorb and retain heat released from the earth increasing the global surface temperature, warming oceans, melting arctic ice, and causing sea level rise. CO_2_ emissions, which were 80% of the released GHGs in 2019, have increased the atmospheric CO_2_ concentration from 331 ppm (1975) to 416 ppm (current), raising the global average surface temperature by nearly 1 °C.^1^ Maintaining the current GHG emission profile will lead to an increase of the global surface temperature by more than 1.5 °C in the near future.^1,2^ Therefore, it is essential to develop robust technologies to limit future emissions of GHGs and reverse the already existing damage.

Direct air capture (DAC) technologies for the removal of CO_2_ from the atmosphere (negative emissions) and carbon capture technologies for large point sources (avoided emissions) such as coal-fired power plants are proposed technologies to address the continuously increasing greenhouse gas emissions.^3^ Considering the large amount of CO_2_ present in the atmosphere and the goal of limiting the global surface temperature increase below 2 °C, quickly developing and deploying DAC processes, alongside other negative emission technologies,^4^ is important to climate stabilization. To this end, developing practical sorbent materials with high CO_2_ selectivity, adsorption capacity, and cyclic stability is necessary.^1,5,6^

Amine-functionalized sorbents are components of promising DAC technologies that are in the early stages of commercialization due to their high CO_2_ selectivity, moderate regeneration energies, high CO_2_ adsorption capacities, and capability to perform under dry and humid conditions.^7,8^ Poly(ethylenimine) (PEI) supported in porous γ-Al_2_O_3_ (PEI/γ-Al_2_O_3_) is a well-known and well-studied supported aminopolymer sorbent in the DAC literature and is the focus of this study.^7,9^ One drawback of amine-functionalized adsorbents is their susceptibility to the loss of their high CO_2_ adsorption capacity when operating continuously over multiple adsorption/desorption cycles in DAC systems. In a typical DAC adsorption–desorption cycle, the sorbents are exposed to ambient air composed of several components such as O_2_, CO_2_, N_2_, H_2_O, and other small atmospheric molecules and particles. These components together with varying process parameters during different parts of the cycle (temperature, pressure, etc.) affect the long-term sorbent stability in a complex way.^10–14^ One main example of this is the copresence of high O_2_ concentration (∼21 mol %) and elevated temperatures associated with sorbent regeneration for short periods of time, which are considered to be the primary opportunity for oxidative degradation. The presence of other components of ambient air such as CO_2_ and H_2_O, in addition to the elevated O_2_ concentration, alongside constantly changing process conditions, leads to an array of factors that may affect the sorbent’s oxidative degradation behavior, mechanism(s), and the resulting longevity of the sorbent.

Studies of materials and processes for DAC to date have mostly focused on improving the CO_2_ adsorption capacities of solid sorbents. While the adsorption capacity is an important factor, several studies have indicated that a sorbent material that can be used for thousands of adsorption/desorption cycles would substantially reduce overall DAC technology costs.^9^ Of the studies on sorbent deactivation to date, most have focused on investigating the effects of exposure to O_2_, H_2_O, or CO_2_ at elevated temperatures.^15–17^ Furthermore, high temperatures that may not be directly relevant to the process are often used to accelerate the deactivation kinetics. The most explored deactivation paths include CO_2_-induced deactivation in simulated flue gas and oxidative degradation in simulated air (21% O_2_ balance N_2_, dry, CO_2_-free). In 2012, Heydari-Gorji and Sayari^10^ investigated the thermal, oxidative, and CO_2_-induced deactivation of PEI-impregnated mesoporous silica (SBA-15) with a platelet particle morphology and short pore channels under inert gas, simulated air, simulated flue gas, and various CO_2_/O_2_/N_2_ conditions. They found that the presence of humidity helped mitigate CO_2_-induced deactivation using a 5% CO_2_/N_2_ mixture. Similarly, in prehumidified CO_2_/O_2_/N_2_ mixtures, they observed improved sorbent stability, suggesting that CO_2_ and/or H_2_O protected them against oxidative degradation.^10^ In a follow-up work by the same group, CO_2_-induced deactivation under dry cyclic conditions using a flow of pure CO_2_ both for adsorption at 50 or 100 °C and desorption at 130–160 °C was explored.^11^ Several amine-grafted MCM-41 and wet impregnated PEI (branched and linear) mesoporous silica sorbents were explored to understand the reaction mechanism(s) and products formed. Both studies suggested urea formation as a result of CO_2_-induced deactivation at elevated temperatures and atmospheric pressure. As such, reaction pathways for open chain and cyclic urea formation were explored by others.^10,11^ Didas et al. explored the thermal, oxidative, and CO_2_-induced deactivation of primary amine-grafted mesoporous silica (SBA-15) adsorbents with alkyl linker lengths varying from methyl to propyl under pure CO_2_ at 135 °C. The ethyl and propyl linkers showed better resistance to oxidative and thermal deactivation; both, however, were vulnerable to CO_2_-induced deactivation.^12^

While these studies helped build foundational knowledge of sorbent deactivation, mainly on CO_2_-induced deactivation, they mostly explored flue gas CO_2_ capture conditions where the CO_2_, H_2_O, and O_2_ concentrations differ from DAC. As such, the roles of CO_2_, H_2_O, and O_2_ in sorbent stability might not be directly transferable to the DAC conditions where the concentrations are different and temperatures are lower. Notably, a recent study from our group on the impact of atmospheric humidity on the stability of the PEI/Al_2_O_3_ sorbent showed that H_2_O plays a significant role in accelerating degradation reactions on some sorbents, which is contrary to other reports in literature, as briefly mentioned above.^15^ To this end, a broad understanding of the impact of ambient air components (H_2_O/CO_2_/O_2_/N_2_) as they appear in the environment on sorbent degradation needs further in-depth exploration.

In this work, we investigate the environmental parameters that influence sorbent stability that have not been fully explored in the DAC literature. Specifically, we assess the impact of incorporating dry and humid CO_2_ in air on the stability of a model aminopolymer sorbent (PEI/Al_2_O_3_) and its influence on the oxidative degradation behavior at intermediate temperatures (70 °C) directly relevant to the CO_2_ desorption process. Furthermore, we explore the effect of humidity on the sorbent stability in the presence and absence of CO_2_ at the same temperature. In situ spectroscopic, thermal, and elemental characterizations of pristine and oxidized sorbents are performed using horizontal attenuated total reflection infrared (HATR-IR) spectroscopy, alongside thermal analysis via thermogravimetric analysis to achieve the experimental goals. Metadynamics simulations are performed to gain further insight into the role of CO_2_ during the oxidation degradation. The knowledge gained from this study will enable further identification of aminopolymer sorbent deactivation pathways and products.

## Experimental Methods

### Materials and Sorbent Synthesis

Aminopolymer sorbents were synthesized by wet impregnation of PEI (branched, 800 g/mol, Sigma-Aldrich) onto a mesoporous gamma-alumina (CATALOX HP 14/15 γ-Al_2_O_3_, Sasol) support (surface area 136 m^2^/g and pore volume 0.95 ± 0.05 cm^3^/g). A branched 45 wt % PEI/γ-Al_2_O_3_ sorbent was used for both infrared spectroscopic analysis and thermal analysis studies. Commercially available alumina (γ-Al_2_O_3_) is used in this study because it is a well-studied support in amine-functionalized sorbents in DAC due to its high surface area and pore volume and offers a more accessible pathway for scale-up and deployment due to the availability of alumina wash-coated monoliths.^14^ Furthermore, it has a higher hydrothermal stability compared to that of the other commercially available supports such as SiO_2_, mainly due to their crystallinity and lower hydrophilicity.^18,19^ Hydrothermal stability is essential considering the practical use of steam regeneration in large-scale DAC plants/processes.^14,20^ γ-Al_2_O_3_ has also been reported to have lower environmental impact in comparison to that of other support materials studied for DAC applications.^21^

N_2_ physisorption was used to characterize the pore volume and pore size of the sorbent (PEI/γ-Al_2_O_3_) and the support (γ-Al_2_O_3_). N_2_ physisorption was performed on a Micromeritics TriStar II 3020 Version 3.02 at 77 K using 100–150 mg of sorbent. The samples were pretreated under a vacuum at 100 °C (γ-Al_2_O_3_) or 60 °C (PEI/γ-Al_2_O_3_) for 10 h prior to the adsorption measurement. The pore size distribution was calculated using the Barrett–Joyner–Halenda method.^22^ Elemental analysis of the fresh and deactivated sorbents was carried out by Atlantic Microlabs to determine the CHN content of the samples before and after various treatments. Specifically, the C/N and H/(C + N) ratios were calculated to understand the evolution of the elemental composition as the sorbent deactivates.

Thermogravimetric combustion analysis was used to determine the total organic content (PEI) of the sorbent. Organic combustion analysis was performed on a thermogravimetric analyzer (TA Instrument Q550) by heating the sample from room temperature to 700 °C with a ramp rate of 10 °C/min under air (Airgas, UZG UZ300, 21% O_2_ balance N_2_). The weight loss of the sorbent from 160 to 700 °C was taken as organic content.

### CO_2_ Adsorption Experiments

CO_2_ adsorption experiments were conducted using a thermogravimetric analyzer (TA Instrument Q500) with 22 ± 0.2 mg of sample on a 50 μL platinum pan to investigate the CO_2_ adsorption capacity. To pretreat the sample, He (Airgas UHP300) gas at 90 mL/min was flowed, and the temperature was increased to 100 °C from room temperature at a ramp rate of 10 °C/min and maintained for 1 h. Then, the temperature was decreased to 30 °C at a ramp rate of 10 °C/min. Once the system equilibrated at 30 °C, the gas was switched to 400 ppm of CO_2_ (balance He) at the same flow rate and held for 3 h to allow for CO_2_ adsorption. After 3 h of adsorption, the gas was switched back to He (Airgas UHP300) at 90 mL/min, and the temperature was increased to 100 °C at a ramp rate of 10 °C/min to desorb the CO_2_. The temperature was held at 100 °C for 1 h to allow the complete desorption of CO_2_. At the end of the desorption, the temperature was reduced to 30 °C at a ramp rate of 10 °C/min to cool the instrument and prepare for the next run.

### Thermal Analysis Experiments

Oxidation experiments were performed using a thermogravimetric analysis–differential scanning calorimetry TGA-DSC (TA Instruments Q600) at 70 °C and 1 atm using 28−35 mg of sorbents (45% PEI/γ-Al_2_O_3_) on a 40 μL alumina ceramic pan. Prior to performing the deactivation study, the sample was purged with N_2_ (Airgas UHP 99.999%) at a flow rate of 100 mL/min at room temperature for 25 min followed by pretreatment at 100 °C for 1 h to remove the adsorbed moisture and CO_2_. After the pretreatment, the temperature was reduced to 70 °C at a ramp rate of 5 °C per minute. Then, the temperature was held at 70 °C (isothermal), and the gas was switched to air (Airgas, UZG UZ300, 21% O_2_ balance N_2_) (CO_2_-free air), 0.04% CO_2_/21% O_2_ balance N_2_ (0.04% CO_2_-air), or 0.04% CO_2_ balance N_2_ (0.04% CO_2_–N_2_) at 100 mL/min for the specified period of deactivation. For humid deactivation experiments, the deactivating gases were prehumidified by flowing through a saturated K_2_CO_3_ solution at a temperature of 23 °C, which provides ∼43% relative humidity (RH). The RH of the gas stream was monitored using a LI-COR 850 H_2_O/CO_2_ analyzer and was constant throughout the experiment. When the deactivation experiment was completed, the gas was switched back to N_2_, and the temperature was lowered to 25 °C to limit further deactivation of the sorbent.

### In Situ ATR-IR Spectroscopy

In situ IR experiments were conducted using a Thermo Scientific Nicolet 8700 HATR-IR) spectrometer with a ZnSe crystal cell to observe the effect of oxidation on the PEI/γ-Al_2_O_3_ sorbent, tracking functional group formation due to the various thermal treatments. For each experiment, a slurry of 45 wt % PEI/Al_2_O_3_ in methanol was prepared by stirring the mixture overnight at room temperature (∼23 °C). After the slurry was well mixed, some of the methanol was removed by rotary evaporation at 50 °C and 218 mTorr for about 2 min. The remaining PEI/Al_2_O_3_/methanol slurry was added dropwise to an ATR-IR ZnSe crystal to form an ∼10 μm thin film. The thin film was purged under N_2_ (Airgas UHP 99.999%) at 100 mL/min for about 20 min at room temperature (∼23 °C) and preheated at 100 °C for 1 h to remove the methanol solvent and the other adsorbed species (such as H_2_O and CO_2_) acquired during sample storage or preparation steps. The thickness of the film was confirmed by scanning electron microscopy (SEM) imaging of the PEI/Al_2_O_3_-coated ZnSe crystal. After pretreatment, the temperature was reduced to 70 °C, and the samples were exposed to a continuous flow of deactivating gas [CO_2_-free air (Airgas UZG UZ300, 21% O_2_ balance N_2_), 0.04% CO_2_/21% O_2_ balance N_2_, or 0.04% CO_2_ balance N_2_] at 100 mL/min, while the cell temperature was held at 70 °C for 3 days. The HATR-IR spectra shown in this study were produced by subtraction of the spectrum of PEI/Al_2_O_3_ at time = 0 at 70 °C under the respective gas mixtures. As such, the spectra shown in the figures (with both positive and negative bands) are a result of the changes in the sample due to exposure to the experimental conditions. A period of 3 days of deactivation was chosen based on the sorbent deactivation data obtained from the thermal analysis study. Peak deconvolution analysis was performed using the SciPy optimize curve fit function in Python to distinguish among the peaks collected from the in situ HATR-IR experiments that are of interest and overlap with each other.

### First-Principles Metadynamics Simulations

Ab initio molecular dynamics (AIMD) and metadynamics simulations were performed with the Vienna ab initio simulation package (VASP), version 5.4.4,^23^ using the projector-augmented wave treatment of the core–valence interactions^24,25^ with the Perdew–Burke–Ernzerhof (PBE) generalized gradient approximation^26^ for the exchange–correlation energy. The DFT-D3 method of Grimme et al. was employed for the van der Waals-dispersion energy corrections.^27^ Triethylenetetramine (TETA) was used in all the simulations as a small-molecule surrogate for PEI. A 15 Å × 15 Å × 15 Å cubic supercell was used to accommodate TETA and the CO_2_-bound TETA molecules, and the Brillouin zone was sampled at the Γ-point only. 10 ps of NVT AIMD simulations at 343 K were run to pre-equilibrate structures before initiating the metadynamics simulations. Coordination numbers as defined in VASP were used as the collective variables (CVs). To reduce the sampling errors, a fine set of Gaussian hill parameters were used: height 0.0025 eV and width 0.02 CV unit. The Gaussian hills were added to the underlying potential energy surface every 20 fs. Metadynamics simulations were terminated after the CVs crossed from the reaction basin into the target product basin following a previously reported protocol.^28^ The free-energy barrier was computed by summing the amounts of bias potentials accumulated in the reactant basin.

## Results and Discussion

### Thermal Analysis

Oxidative and thermal degradation of the 45% PEI/γ-Al_2_O_3_ sorbent: a model PEI/Al_2_O_3_ sorbent with a PEI loading of 45 wt % and about 98% pore fill (0.028 cm^3^/g) was used for the thermal analysis study. The physical characteristics of the sorbent are determined using N_2_ physisorption. TGA-DSC was used to follow the weight loss and heat flow of 28–35 mg of the 45 wt % PEI/γ-Al_2_O_3_ sorbent as a function of time. The sorbent was exposed to CO_2_-free air (21% O_2_) for 3, 7, 10, and 14 days and to 99+% N_2_ for 3, 7, and 14 days continuously at 70 °C for oxidative and thermal degradation experiments, respectively. The weight loss recorded at 70 °C was assumed to be a result of PEI deactivation. Figure S1 shows the thermogravimetric combustion measurement of the model sorbent (45 wt % PEI/γ-Al_2_O_3_). The CO_2_ adsorption capacities of the fresh and oxidized samples were determined using 400 ppm of CO_2_ balance He stream. Sorbent deactivation is defined as the percent loss in the CO_2_ adsorption capacity (mmol of CO_2_/g of sorbent) from a fresh sorbent after exposure to a specified gas mixture and a deactivation period at 70 °C. The CO_2_ adsorption capacity of a fresh sorbent was 1.34 mmol of CO_2_/g sorbent.

**Figure 1:**
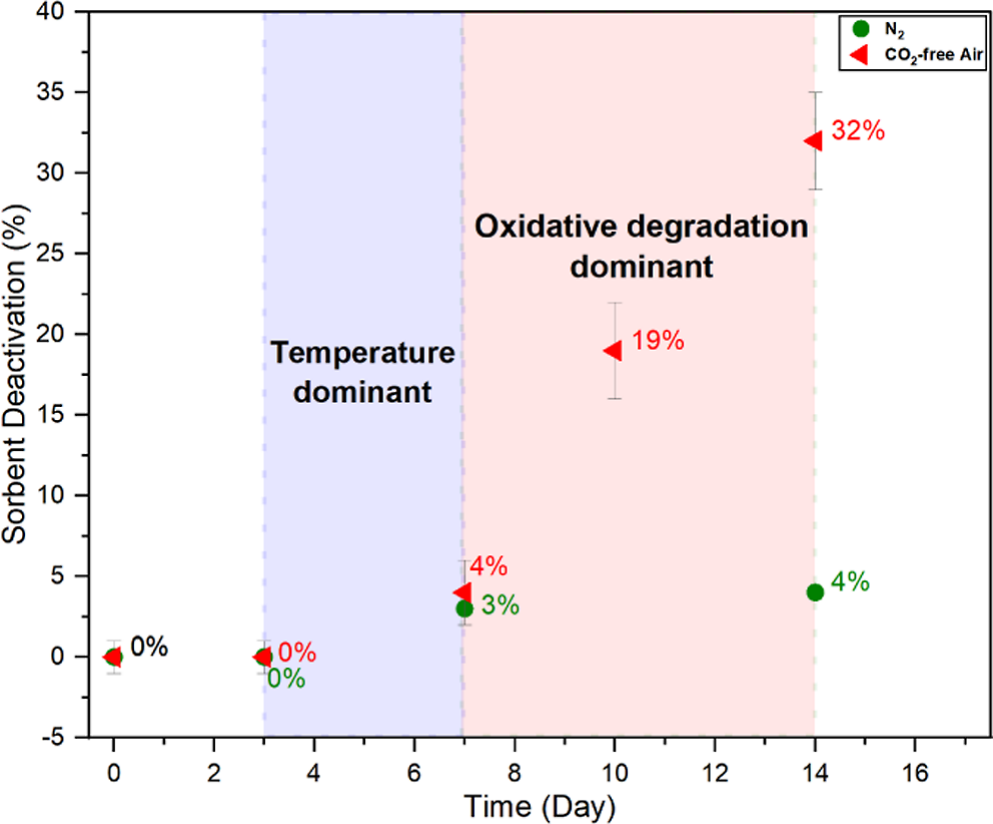
Oxidative and thermal degradation of the 45% PEI/γ-Al_2_O_3_ sorbent at 70 °C.

As the results in Figure 1 show, oxidative and thermal degradation appeared to have similar impact on the loss of adsorption capacity until day 7 (4% vs 3% loss in capacity, respectively). We assign this region of the degradation as “temperature dominant” as most of the impact can be attributed to thermal effects. After day 7, there was more significant sorbent deactivation under oxidative degradation conditions compared with the thermal-only conditions. This region is assigned as the “oxidative degradation”-dominant region.

**Figure 2:**
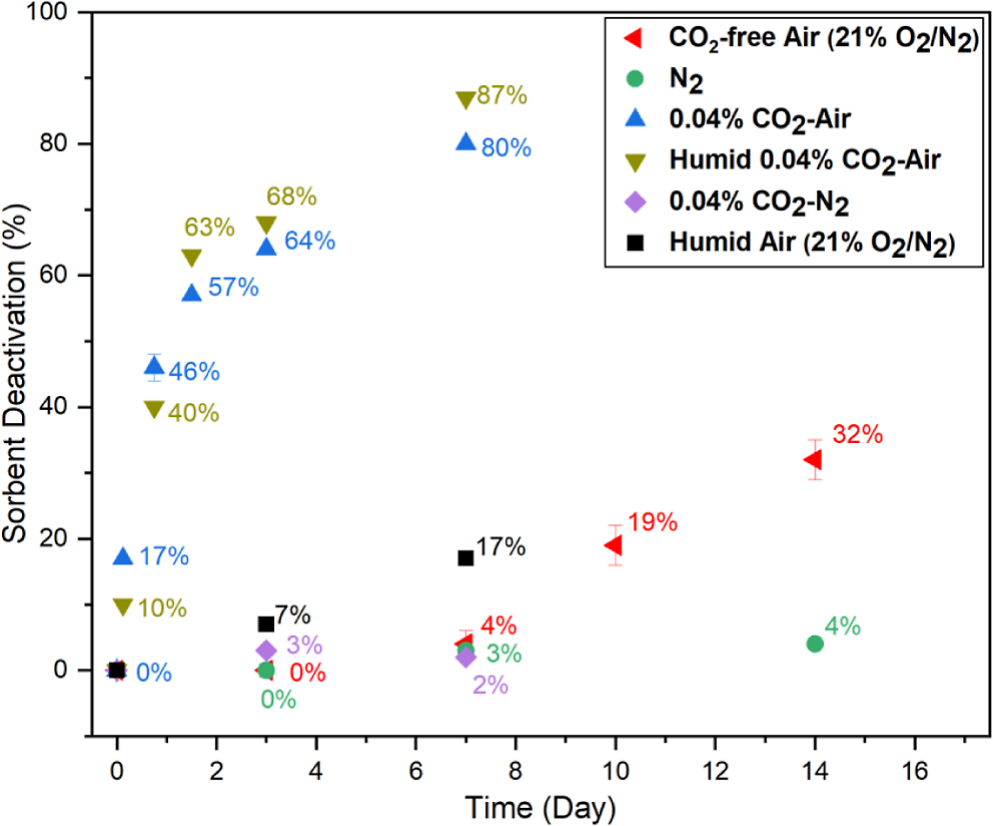
Deactivation of the 45% PEI/γ-Al_2_O_3_ sorbent under CO_2_-free air (for 3, 7, 10, and 14 days), under N_2_ (for 3, 7, and 14 days), under 0.04% CO_2_-air (for 3 h, 18 h, 36 h, 72 h, and 7 days), and 0.04% CO_2_ balance N_2_ environments at 70 °C for 3 and 7 days.

Building on these results as a baseline, we then investigated the presence of CO_2_ and H_2_O together and separately. As shown in Figure 2, after 3 and 7 days of continuous exposure to 0.04% CO_2_–N_2_ flow, the loss in the CO_2_ adsorption capacity was minimal (3 and 2%, respectively; purple diamonds), indicating limited deactivation. On the contrary, under 0.04% CO_2_-air, notable loss in CO_2_ adsorption capacity was observed, even in the early stages of deactivation (17% loss after 3 h, blue triangles). Though the deactivation rate is moderate, it eliminates the induction period observed under oxidative and thermal degradation conditions in the absence of CO_2_ (Figure 1). After 3 h, the deactivation continued to increase, reaching 80% loss after 7 days. Thus, the combination of CO_2_ and O_2_ leads to more rapid deactivation than with either species alone.

In a humid environment [43% RH; olive triangles], a stream of 0.04% CO_2_-air yielded similar deactivation behavior as the dry 0.04% CO_2_-air mixture. The initial deactivation rate appeared slightly accelerated under dry conditions and slightly slower at longer times, relative to humid conditions, but these apparent differences are likely within the margin of experimental error. The data in Figure 2 show that in the absence of CO_2_, the differences between dry and humid environments were magnified (dry, red triangles; humid, black squares), with higher deactivation under humid conditions after 7 days. This difference suggests that CO_2_ has a bigger impact on oxidative degradation than H_2_O under the continuous deactivation conditions explored, with H_2_O enhancing degradation in the absence of CO_2_. The enhancement of oxidative degradation in the presence of H_2_O vapor is consistent with the impact of H_2_O in accelerating oxidative degradation reported in our recent work.^15^

The above results (Figure 2) show the implications of the copresence of CO_2_ and O_2_, as they appear in ambient air, on the stability of the amine-based sorbents at an intermediate temperature. As briefly mentioned above, these results differ from what has been reported in the literature for different amine sorbents under slightly different conditions. Previously, the presence of CO_2_ in O_2_-containing streams was reported to improve the stability of the amine-based sorbents based on the fact that the amine species react faster with CO_2_ compared to O_2_, forming carbamate and bicarbonate species that reportedly enhance stability.^10^ In the work of Heydari-Gorji and Sayari,^10^ the thermal, oxidative, and CO_2_-induced deactivation of PEI (branched and linear)-impregnated mesoporous silica (SBA-15) sorbents with the platelet particle morphologies and short pore channels was explored. In their specific study pertaining to the copresence of CO_2_ and O_2_, a PEI/SBA-15 sorbent was deactivated continuously under prehumidified CO_2_/O_2_/N_2_ streams at different concentrations (1–20%/10.5–17%/balance N_2_) and temperatures ranging from 50 to 120 °C for 30 h. See Table S1 for tabulated values. The study reported no significant deactivation below 100 °C for all of the CO_2_/O_2_/N_2_ mixtures. At 100 °C, 70% CO_2_ uptake loss was obtained for the 1%/17%/82% (CO_2_/O_2_/N_2_) gas mixture after 30 h, and the uptake loss decreased significantly as the CO_2_ concentration increased to 20% (2.6% loss Table S1).^10^ In contrast, in the current study, the 45 wt % PEI/Al_2_O_3_ sorbent deactivation treatments at 70 °C in both dry and humid 0.04% CO_2_-air mixtures yielded noticeable losses in CO_2_ adsorption capacity even after 3 h of deactivation (loss of 17% for dry and 10% for humid). Previously, the presence of H_2_O was reported to have a positive effect during the oxidative degradation, retarding oxidation;^10^ however, as seen in Figure 2 and as reported in our recent work, the outcome in our system is reversed.^15^ When rationalizing these differing results, it is important to note the key differences between the two studies, which include the deactivation temperature (50–120 °C for the Heydari-Gorji and Sayari study and 70 °C for this study), CO_2_ concentration (1–20% for the Heydari-Gorji and Sayari study and 0.04% for this study), CO_2_ uptake temperature (75 °C for the Heydari-Gorji and Sayari study and 30 °C for this study), and support type (laboratory-synthesized ordered mesoporous silica vs disordered, commercial mesoporous alumina). Further discussion comparing and contrasting our results to the literature will be presented after additional analysis in the forthcoming sections.

### Spectroscopic Analysis

**Figure 3:**
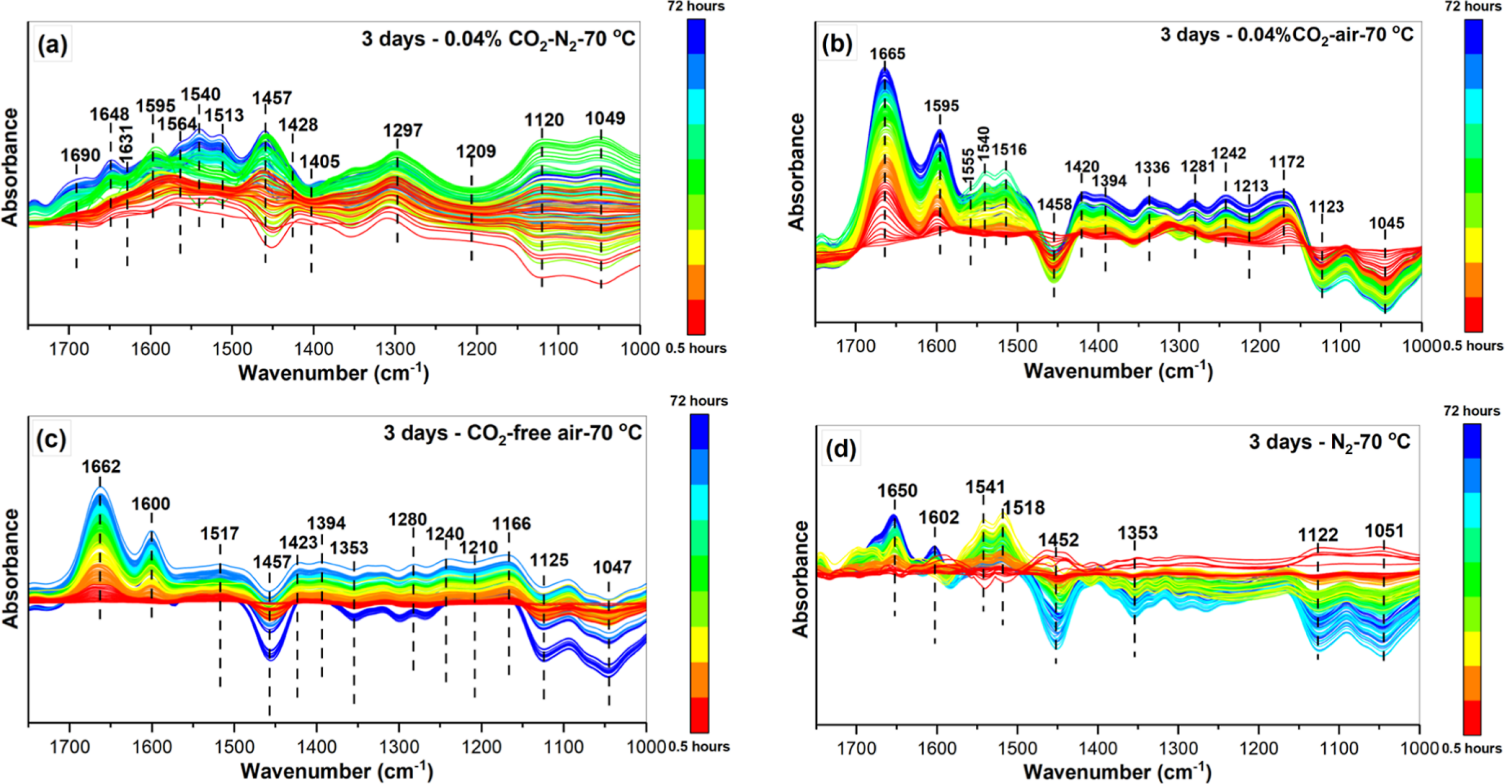
IR spectra (1750–1000 cm^–1^) of PEI/Al_2_O_3_ deactivation under (a) 0.04% CO_2_–N_2_, (b) 0.04% CO_2_-air, (c) CO_2_-free air, and (d) 100% N_2_ for 3 days (72 h) at 70 °C. (Full spectra (3500–1000 cm^–1^) in Figure S2.

To investigate the accelerated sorbent deactivation in the copresence of CO_2_ and O_2_, in situ HATR-IR spectroscopy experiments were performed using a 45 wt % PEI/Al_2_O_3_ sample as a model sorbent. Experiments were conducted using different gas/vapor compositions, including dry CO_2_-free air, 0.04% CO_2_-air (21% O_2_ balance N_2_), and 0.04% CO_2_–N_2_ at 70 °C for 3 days (Figure 3 and S2). Complementing the TGA studies above, these experiments allow the elucidation of molecular level changes due to PEI/Al_2_O_3_ sorbent deactivation under the conditions described above and the investigation of possible changes in the PEI/Al_2_O_3_ structure. The thermal analysis results given above indicate significant deactivation (∼64%) under 0.04% CO_2_-air after 3 days and no major deactivation under CO_2_-free air, 0.04% CO_2_–N_2_ and N_2_ until after day 7. As such, all the IR spectroscopy experiments were conducted for 3 days by collecting spectra every 30 min. Figures 3 and S2 show the absorption spectra of PEI/Al_2_O_3_ over 3 days at 70 °C under the four experimental conditions.

The main characteristics of a PEI/Al_2_O_3_ sorbent in the infrared spectra are two N–H stretching bands of primary amines (3400–3300 and 3330–3250 cm^–1^), one of secondary amines (3350–3310 cm^–1^), C–H stretching bands (2950–2720 cm^–1^), an N–H bending band 1605–1590 cm^–1^, a C–H bending band ∼1454 cm^–1^, and C–N stretching bands at ∼1125 and ∼1050 cm^–1^.^29,30^

In the early phase of sorbent deactivation under 0.04% CO_2_–N_2_ at 70 °C, the adsorbed CO_2_ dominated the difference spectra, and the thermal deactivation of PEI/Al_2_O_3_ should contribute only minimally to the spectral changes under these conditions (see Figure 1). Figure 3a shows bands of species such as the COO^–^ asymmetric stretch (∼1565 cm^–1^), NH_3_^+^ symmetric deformation (∼1540 cm^–1^), COO^–^ symmetric stretch (∼1428 cm^–1^), C–N stretching band (∼1405 cm^–1^), N–COO (∼1330 cm^–1^), NCOO^–^ skeletal vibration (∼1297 cm^–1^), and NH_2_^+^ at 1631 cm^–1^, which indicate the formation of ammonium carbamate ion pairs in the early stages of the experiment and their growth in intensity with time.^30–43^ In CO_2_ capture studies using amines like PEI, the dominant species that forms due to the interaction of CO_2_ with primary and secondary amines under dry conditions is the carbamate species.^31,33,43–46^

Figure 3a–d also shows the bands in the region of 1666 cm^–1^ that increase in intensity as the treatment time increases. This band has been discussed in the literature as a characteristic of amine-related oxidation products as a result of exposure to O_2_ and has been assigned to the vibrations of C=N (imines) and/or C=O (carbonyl) groups.^29,39,41,47,48^ A recent study from our group on the oxidative degradation of the PEI/γ-Al_2_O_3_ sorbent at high temperatures illustrated the individual contributions of the imine and carbonyl species to the band in the region around 1666 cm^–1^ for the first time.^15^ The study links the C–N bond cleavage events to the formation of carbonyl and imine species, new primary amine species, and other low-molecular-weight amine products.^15^ To briefly discuss the proposed mechanism(s), in dry conditions, degradation begins with the formation of radical species (α- or β-amino alkyl radicals) due to thermal stress, which leads to chain relaxation and breakage,^15^ or due to metal impurities within the sorbent that catalyze free radical formation.^15,49^ In the presence of O_2_, the radical species react with O_2_ forming peroxyl radical species (α- or β-amino peroxyl radicals). The peroxyl radicals extract hydrogen from the PEI chain, forming hydroperoxide species (ROOH), which decompose to form carbonyl species in the PEI chain.^15^ The formation of the carbonyl species requires the cleavage of the C–N bond, and depending on the location of the C–N bond (terminal primary amine, terminal secondary amine, or secondary amine along the PEI chain), volatile organic products (e.g., ammonia and 2-aminoacetaldehyde) and new primary amine species can form.^15,50^

**Figure 4:**
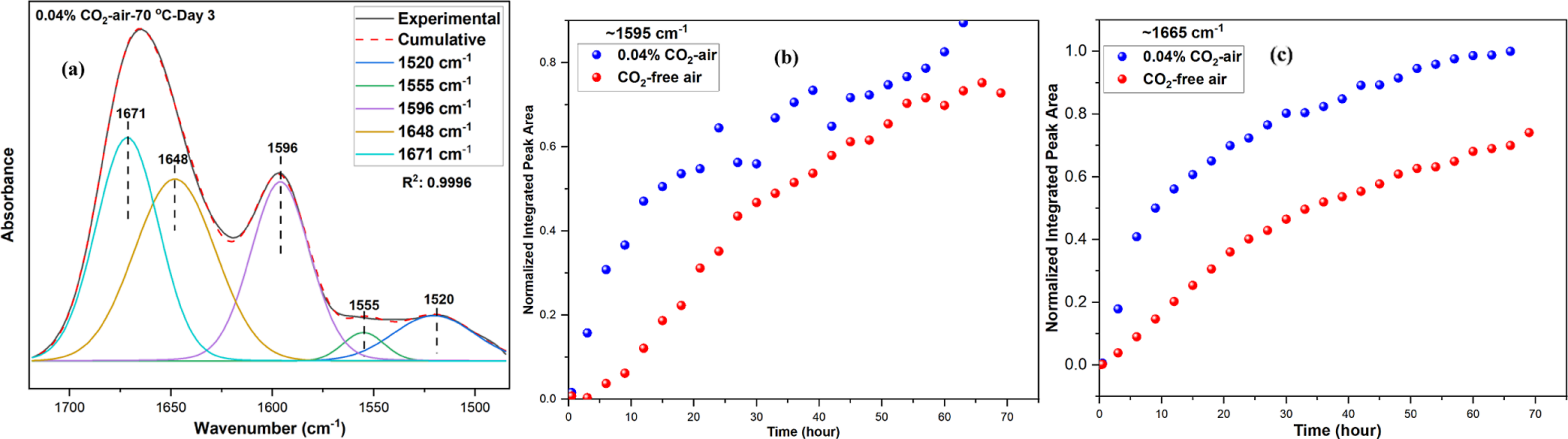
(a) Deconvoluted spectra of 0.04% CO_2_-air at the 72nd hour (end of day 3) from 1720 to 1485 cm^–1^. Integrated peak areas of (b) ∼ 1595 cm^–1^ (N–H bending band) and (c) ∼1666 cm^–1^ (C=O/C=N bands) under 0.04% CO_2_-air and CO_2_-free air.

A simplified deconvolution analysis was performed in the region between 1720 and 1485 cm^–1^ to distinguish the contributions of the imine and carbonyl species to the 1665 cm^–1^ band under 0.04% CO_2_-air conditions. Figure 4a shows the results of the deconvolution analysis under the 0.04% CO_2_-air condition.

**Figure 5:**
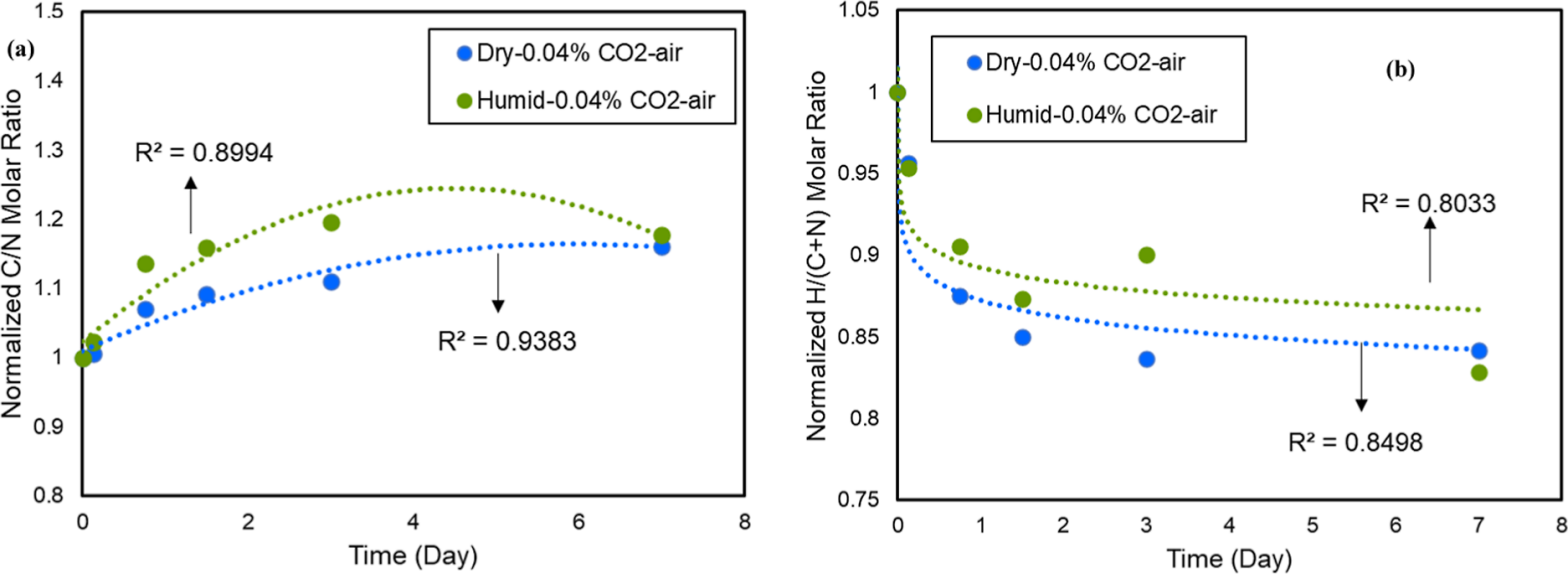
(a) C/N (b) H/(C + N) molar ratio of the deactivated 45 wt % PEI/γ-Al_2_O_3_ sorbent as a function of time under dry 0.04% CO_2_-air and humid 0.04% CO_2_-air.

Two peaks (1671 and 1648 cm^–1^) contributed to the 1665 cm^–1^ band, while only one peak contributed to the 1595 cm^–1^ band. The band ∼1595 cm^–1^ represents an N–H bending (N–H deformation) mode.^15,29,42^ The N–H bending/deformation vibration bands appear in the region between 1650 and 1580 cm^–1^ for primary and secondary amine species.^29^ Furthermore, in liquid amines, the N–H bending band gives rise to a shoulder/overtone band near the N–H stretching regions that can be seen from the band around 3135 cm^–1^ in Figure S2a–d.^29^ As briefly discussed above, the increased intensity of the N–H bending band has been reported in our recent work as an indication of the formation of new primary amine species as a result of the C–N bond cleavage near the secondary amine sites.^15^ Similarly, Figure 3b,c shows an increase in the intensity of the bands around 1595 cm^–1^ with time for 0.04% CO_2_-air and CO_2_-free air conditions. The integrated area of the N–H bending band (∼1595 cm^–1^) for the sample treated in 0.04% CO_2_-air was larger than that for the sample under CO_2_-free air (Figure 4b). This result suggests that the formation of new primary and secondary amine species and C–N bond cleavage reactions occurs more frequently in the copresence of CO_2_ and O_2_, which agrees well with the thermal analysis results shown in Figure 2, where deactivation of the PEI/γ-Al_2_O_3_ sorbent is more significant under a 0.04% CO_2_-air environment compared to that under a CO_2_-free air. The disproportional loss of the hydrogen and nitrogen species with respect to carbon species is shown by the C/N and H/(C + N) molar ratios in Figure 5a,b during the deactivation of the sorbent under both dry and humid 0.04% CO_2_-air conditions. The higher loss of N and H content compared to that of C content is an additional sign of the C–N bond cleavage, as indicated by the spectra in Figure 3b. Figure S3 also shows the loss of hydrogen and nitrogen species and an increase in carbon species under both dry and humid conditions.

The increase in the intensity of the band ∼1665 cm^–1^ shown in Figure 3b,c under CO_2_-free air and 0.04% CO_2_-air environments can be attributed to the formation of carbonyl and imine species based on the deconvolution results in Figure 4a and the evidence for the C–N bond cleavage reactions, as also indicated by the negative bands at 1123 and 1045 cm^–1^ for 0.04% CO_2_-air and at 1125 and 1049 cm^–1^ for CO_2_-free air. The 1671 cm^–1^ and the 1648 cm^–1^ bands, which are the two peaks that contribute to the 1665 cm^–1^ band, can be attributed to the carbonyl species (C=O) and imine species (C=N), respectively, based on the presence of the 1648 and 1650 cm^–1^ bands that indicate imine species formation under 0.04% CO_2_–N_2_ and N_2_ environments (O_2_ free) (Figure 3a,d). Under 0.04% CO_2_–N_2_ and under N_2_, the intensities of the imine bands and the primary amine bands that likely occur due to hydrogen abstraction followed by the C–N bond cleavage are much less pronounced compared to the imine/carbonyl and primary amine band intensities under 0.04% CO_2_-air. This result suggests similar deactivation under 0.04% CO_2_–N_2_ and N_2_ environments and agrees well with the thermal analysis results for the deactivation of the PEI/γ-Al_2_O_3_ sorbent in a 0.04% CO_2_–N_2_ and N_2_ environment at 70 °C, as shown in Figure 2.

The C–N bond cleavage leading to the formation of new primary amine species (as well as other products mentioned above) has been reported as paths of oxidative and thermal degradation at high temperatures in solid amine sorbents.^15,16,50^ As such, these results suggest the possible occurrence of oxidative and thermal degradation under CO_2_-free air and 0.04% CO_2_-air conditions and thermal degradation under 0.04% CO_2_–N_2_ and N_2_ conditions. However, the accelerated PEI/γ-Al_2_O_3_ sorbent deactivation and new primary amine species formation under 0.04% CO_2_-air, as shown in Figures 2 and 4b, indicate the presence of additional reaction mechanism(s) occurring in the presence of CO_2_.

### Metadynamics Simulations

A previous modeling study by Li et al.^16^ used TETA as a molecular proxy for PEI and assessed the kinetic feasibility of C–N bond cleavage in the presence of radicals but without CO_2_ using enhanced sampling, i.e., metadynamics simulations.^16^ To this end, we performed first-principles metadynamics simulations using CO_2_-bound TETA as a molecular proxy to analogously evaluate the kinetics of the C–N bond cleavage of PEI/Al_2_O_3_ under CO_2_-air conditions.

**Figure 6:**
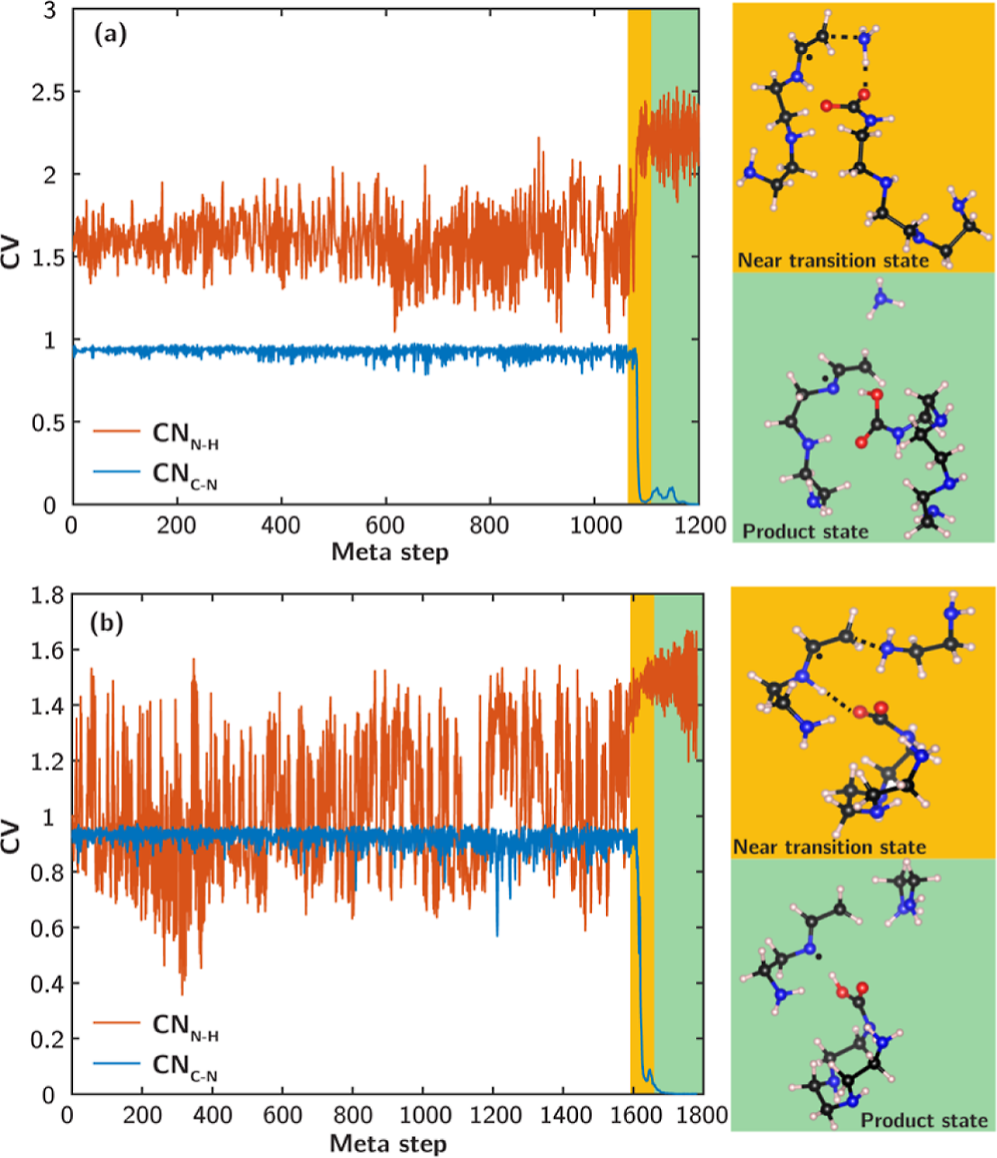
Time evolution of CVs along the trajectories of metadynamics simulations of the CO_2_-catalyzed C–N bond cleavage near (a) the primary amine and (b) the secondary amine. Structural snapshots near transition states and product states are shown. Atom color code: C—black, N—blue, O—red, and H—pink.

To model the interactions between CO_2_ and amines, we constructed bimolecular structural models consisting of one TETA with CO_2_ adsorbed to a primary amine site, carbamic acid. CO_2_ adsorbed as an ammonium carbamate ion pair will behave similarly due to the dynamic equilibrium between a hydrogen-bonded carbamic acid–amine pair and the ammonium carbamate. The adsorbed CO_2_ further interacts with either a primary or secondary amine on another TETA with a preformed alkyl radical, presumably resulting from radical propagation under oxidative conditions. The AIMD-equilibrated structures are presented in Figure 6 and were prepared for metadynamic simulations with a temperature set at 70 °C to replicate the experimental conditions. The metadynamics approach adds periodic biasing potentials to the original potential surfaces along predefined CVs, which discourage the system from revisiting points in the configurational space, resulting in the acceleration of energy barrier crossing for the sampled reactions.^51^ To determine the kinetics of the C–N bond cleavage, two CVs were defined: C–N coordination number (CN_C–N_) and N–H coordination number (CN_N–H_) of amines interacting with the carbamic acid in proximity.

**Scheme 1:**
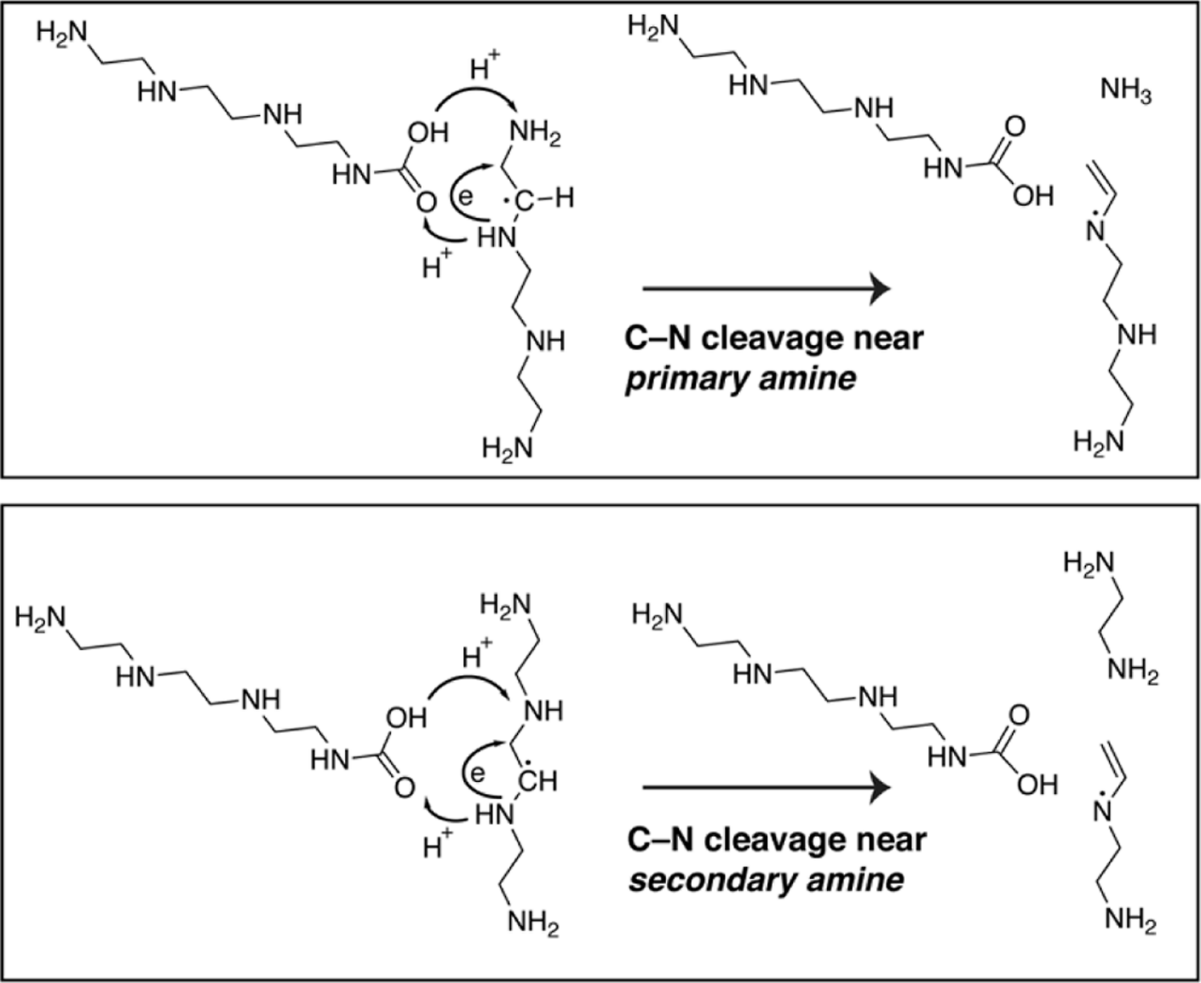
Reaction Pathways of the C–N Bond Cleavage near Primary and Secondary Amines in the Presence of CO_2_

Figure 6 displays the evolution of CVs during metadynamics simulations for the C–N bond cleavage near the primary and secondary amines. The successful cleavage of the C–N bonds is observed in both cases, as evidenced by sharp decreases in the level of CN_C_–_N_ from approximately 1 to 0. Additionally, simultaneous increases in CN_N_–_H_ and the structural snapshots near the transition states demonstrate that for both primary and secondary amines, H transfer occurs from a neighboring amine group to the affected amine, resulting in the formation of NH_3_ and ethylene diamine, respectively. Scheme 1 illustrates that the carbamic acids function as effective H-shuttles, facilitating the proton and electron transfer necessary to cleave the C–N bonds. The products consist of fragments of TETA and regenerated TETA with a carbamic acid tail. The formation of a new primary amine in the case of the C–N bond cleavage near a secondary amine is consistent with the observations from spectroscopic experiments. Significantly, the sum of the biasing potentials indicates that in the presence of CO_2_, both reactions of C–N bond cleavage have free-energy barriers of ∼20 kJ mol^–1^, which are considerably lower compared to that in the absence of CO_2_ (∼90 kJ mol^–1^).^16^ This manifests the strong catalyzing effect of CO_2_ on the C–N bond cleavage kinetics through acid–base interactions, consistent with the accelerated PEI/γ-Al_2_O_3_ sorbent deactivation under 0.04% CO_2_-air conditions.

Based on the spectroscopic, thermal, and elemental analyses and metadynamics simulations performed in this study and the accumulated knowledge in the aminopolymer degradation literature, we rationalize the accelerated deactivation in the copresence of CO_2_ and O_2_. Figure 4b,c shows that the difference in the rate of formation of new primary amines and carbonyl/imine species is not significant for 0.04% CO_2_-air and CO_2_-free air. However, the thermal analysis data in Figure 2 showed that the deactivation under 0.04% CO_2_-air was substantially higher, even at the early stages of deactivation (3 and 18 h). The difference in deactivation between the two conditions suggests that under a CO_2_-free environment, the C–N cleavage process occurs at a slower rate (compared to 0.04% CO_2_-air). Based on the oxidative degradation pathway proposed in our recent work^15^ and the basic autoxidation mechanism,^52^ the slower rate of C–N cleavage is likely due to the substantial energetic barrier for the decomposition of hydroperoxide species to hydroxyl and alkoxyl radical species in the oxidative degradation process.^52^ The deactivation temperature studied here (70 °C), which is much lower than those of prior studies, is likely not high enough to allow the decomposition process to occur rapidly. In the copresence of CO_2_ and O_2_ (0.04% CO_2_-air), CO_2_ reacts with the amine sites forming carbamic acids that can further react with primary or secondary amines in the surrounding region via acid–base interactions. Some of these species become captured CO_2_ in the form of alkyl ammonium carbamates, while some facilitate H-transfer and C–N bond cleavage forming ammonia, primary amine, or secondary amine species, depending on the site of CO_2_ adsorption, as supported by the metadynamics simulations. This acid-catalyzed process requires the presence of alkyl radicals on the PEI chain, where C–N cleavage occurs. Oxygen, which is present in much higher concentration than CO_2_, can then participate in the formation of peroxyl radicals that abstract hydrogen atoms, leading to more alkyl radical species in what appears to be a carbamic acid-catalyzed decomposition reaction. As a result, the carbamic acid-catalyzed deactivation is proposed to provide an alternative pathway for the C–N bond cleavage that bypasses the hydroperoxide decomposition step and subsequently accelerates the deactivation and formation of the various species discussed above.

### Adsorption–Desorption Cycles

Cyclic adsorption–desorption experiments were performed to develop relationships with the continuous deactivation study under dry and humid 0.04% CO_2_/air (21% O_2_ balance N_2_) conditions at 70 °C and to observe sorbent deactivation under close-to-realistic DAC process conditions. A typical cyclic process involves the adsorption of CO_2_ from the atmosphere, consuming most of the process time, followed by the desorption of CO_2_ captured from the atmosphere and finally cooling of the sorbent to the adsorption temperature. During these adsorption–desorption processes, sorbent materials are exposed to conditions and environments that cause deactivation over an extended period of operation. In practice, on occasions where process upsets occur due to the operational malfunctions or for other reasons, additional deactivation factors are introduced, such as the copresence of high temperatures and high oxygen concentrations.

**Figure 7:**
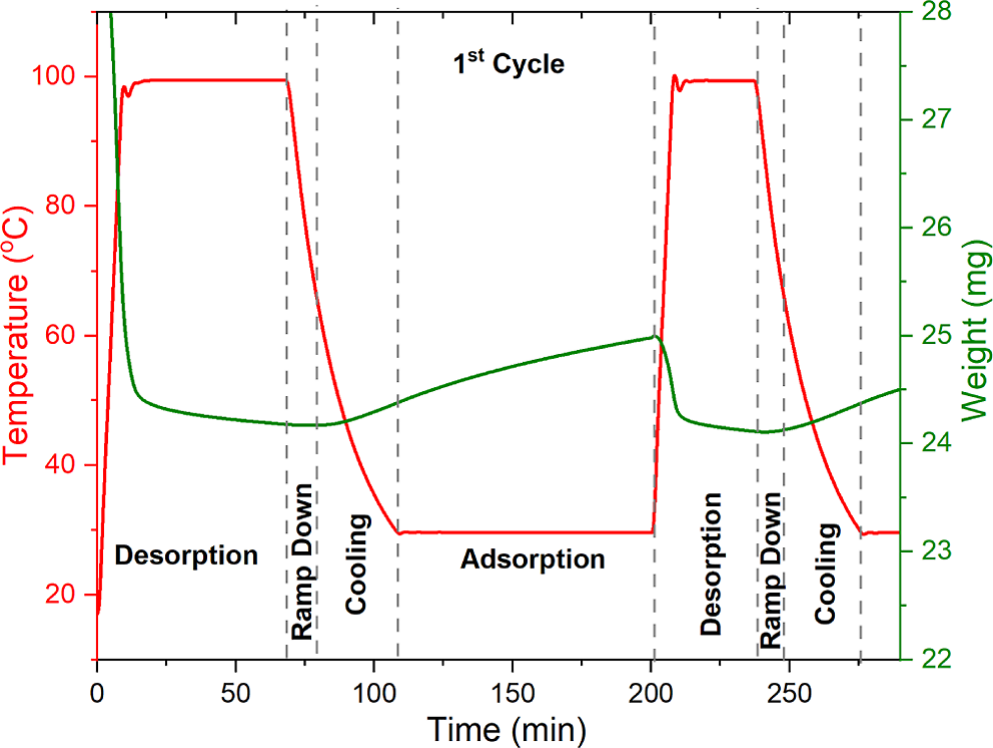
Adsorption–desorption cycle deployed in the TGA cycling.

In these cyclic studies, the 45 wt % PEI/γ-Al_2_O_3_ sorbent was exposed to dry or humid (43% RH or 12 mmol H_2_O/mol air absolute humidity) 0.04% CO_2_-air for 2 h, which represents roughly two-thirds of the laboratory process/cycle time. The captured CO_2_ is desorbed at 100 °C for 30 min under dry N_2_ or humid N_2_ [1.3% RH (absolute humidity of 12 mmol/mol)] to mimic the vacuum-assisted desorption during a temperature–vacuum swing desorption process. After 30 min of desorption, the temperature is ramped down to 70 °C under N_2_ (dry or humid). Following that, the sorbent is cooled to the adsorption temperature of 30 °C under dry or humid 0.04% CO_2_/air (21% w/w O_2_ balance N_2_). This cooling step is typically performed in an inert environment such as N_2_ in prior literature studies of amine sorbent stability; the conditions here more closely mimic those that might be encountered in practical operation. The cooling process takes about 30 min on average in the laboratory but would be more rapid in realistic, low-pressure drop gas–solid contactors. Cooling the sorbent under CO_2_-containing air (atmospheric concentration air) removes the use of inert gases in this step, which is common in the laboratory, and it allows for CO_2_ to be captured during the cooling process. In addition, cooling using atmospheric concentration air eliminates the use of inert gas, reducing the energy and process cost. Figure 7 shows the adsorption–desorption cycle used in this study.

**Figure 8:**
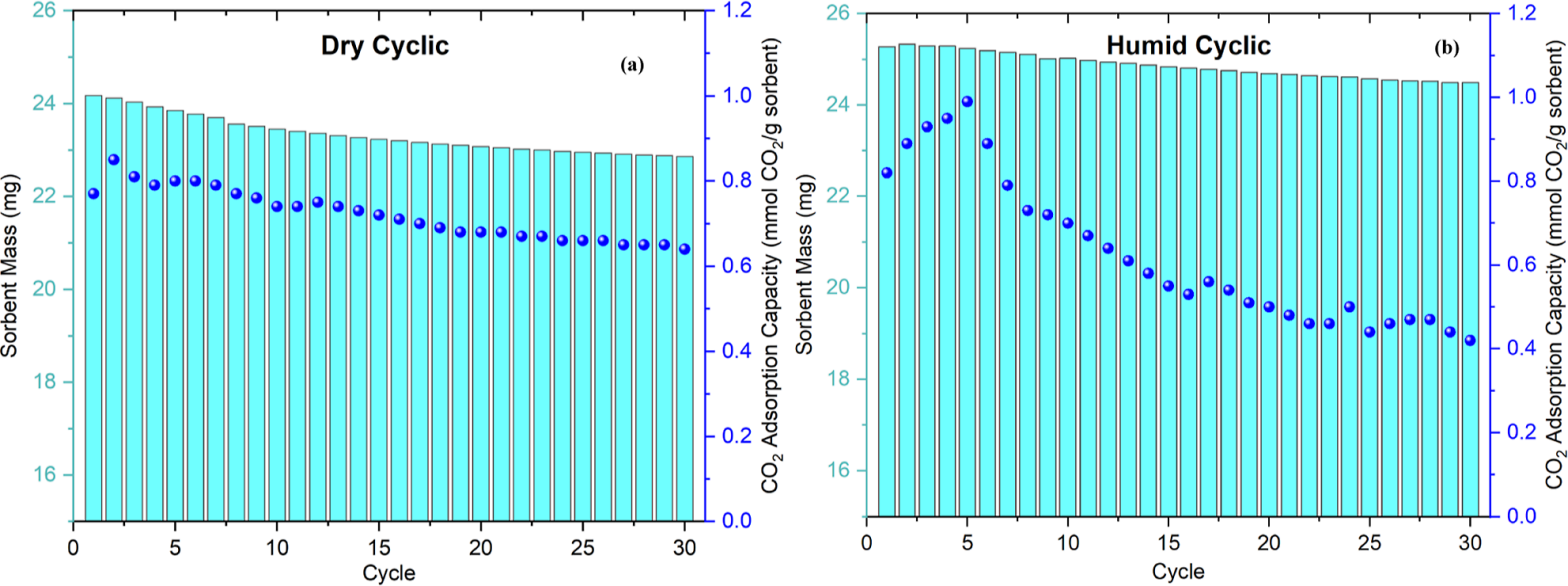
Sorbent mass and CO_2_ adsorption capacity change after 30 cycles under dry (a) and humid (b) 0.04% CO_2_-air.

During the adsorption and cooling process, the sorbent interacts with O_2_ and CO_2_ at low and intermediate temperatures after desorption in flowing N_2_ (simulating vacuum) at higher temperatures. In this cyclic process, the most important time when the oxidative deactivation would be a concern is during the cooling process where the sorbent is exposed to intermediate temperatures under the copresence of CO_2_ and O_2_, leading up to the next adsorption step. The results in Figure 8a,b show that 30 adsorption–desorption cycles result in a CO_2_ uptake loss of 23% under dry and 48% humid conditions as well as a gradual loss in the sorbent mass (∼6% for dry and ∼4% for humid). Consistent with the continuous deactivation study in Figure 2, the copresence of CO_2_ and O_2_ results in noticeable deactivation under cyclic conditions, and the use of consecutive cycles seeks to ascertain if numerous short exposures begin to approach the deactivation of extended exposure times.

Another interesting finding from this study is that during continuous deactivation, the presence of H_2_O only slightly affected the sorbent deactivation in the copresence of CO_2_ and O_2_, as shown in Figure 2. On the contrary, under cyclic conditions, the presence of H_2_O shows considerable deactivation after 30 cycles compared to the dry cyclic condition (about 2× higher). This might be due to H_2_O gaining more access to the PEI domain and radical species in the absence of CO_2_ (desorption and ramp down step), which can lead to accelerated deactivation.^15^ Another possible explanation for the difference in the CO_2_ capacity loss between dry and humid conditions could be the higher CO_2_ adsorption capacity obtained in the first few cycles under humid conditions. The presence of H_2_O during CO_2_ adsorption enhances the amine efficiency of the sorbent (CO_2_ adsorption capability of the sorbent) by accelerating the formation of hydronium carbamate and ammonium carbamate.^37,53^ However, once the sorbent deactivation starts to occur, the presence of humidity plays a reverse role, in which it participates in accelerating the loss of the CO_2_ adsorption capacity of the sorbent^15^ under some conditions. As such, the drop in CO_2_ adsorption capacity after deactivation starts is reduced in dry conditions compared to that in humid conditions. Notably, this result is consistent with our recent finding on the effect of humidity in accelerating the sorbent deactivation that shows 2× faster deactivation in the presence of H_2_O conditions.^15^

It is important to note that the cyclic CO_2_ uptake under humid conditions was determined by performing the same cyclic experiment under humid N_2_ for 15 cycles and then subtracting the H_2_O vapor uptake from that measured in the 0.04% CO_2_-air humid cyclic experiment. During the humid N_2_ cyclic experiment, the H_2_O adsorbed per cycle was around 2.2 mg (shown in Figure S4). In the 0.04% CO_2_-air humid cyclic experiment, this amount will most likely be less than 2.2 mg due to the competitive interaction between CO_2_ and H_2_O vapor with the amine sites during the adsorption step. As such, the CO_2_ uptake reported in Figure 8b for the humid cyclic conditions likely slightly underestimates the actual capacity.

The total time the sample was exposed to elevated temperature, during the desorption step at 100 °C and the cooling step from 100 to 70 °C, sums up to approximately 17 h across the 30 cycles. During these steps, the sample is only exposed to an inert atmosphere, and so, the thermal degradation due to the loss of sorbent mass is the dominant loss mechanism, 6 and 4% for the dry and humid cyclic conditions, respectively.

The total time the sample was exposed to oxygen, during the cooling step from 70 to 30 °C and the following 2 h adsorption step at 30 °C, sums up to approximately 3 days across the 30 cycles. Therefore, it is reasonable to compare the loss in capacity across the cyclic experiment with the 3 day continuous deactivation experiment. Under both conditions, the loss in capacity under the cyclic conditions is lower (dry: 29% vs 64%, humid: 52% vs 68%). This difference in capacity loss shows that continuous exposure exaggerates deactivation. However, continuous deactivation studies can help estimate the lifetime of a sorbent over an extended period of operation if suitable correlations can be developed for real operation. As such, below, we will use the deactivation mechanism(s) presented in Scheme 1 to rationalize the differences in capacity loss between the continuous and cyclic conditions.

One of the primary reaction intermediates in the deactivation of the sorbent, the peroxyl radicals, forms by the reaction of the alkyl radicals with molecular oxygen. The peroxyl radicals form at similar rates at any temperature once the alkyl radicals form and the sorbent is exposed to an O_2_-containing stream.^52,54^ However, the subsequent step of the reaction, the formation of hydroperoxide species, requires the abstraction of a hydrogen atom from a C–H bond, and this reaction is temperature-dependent. At higher temperatures, more energy is available to overcome the activation energy required for the hydrogen abstraction.^52,54^ Similarly, the rate of decomposition of the hydroperoxide species to alkoxyl and hydroxyl radical species increases with temperature.^52,54^ As such, during the cooling (from 70 to 30 °C) and adsorption step (at 30 °C) of the cyclic process, it is expected to have a lower concentration of hydroperoxide species and that the decomposition of hydroperoxide species occurs at a slower rate compared to continuous deactivation cases at 70 °C. It is also important to note that during the cyclic process, the limited coexistence of O_2_ and heat compared to the continuous deactivation likely plays a role in maintaining sorbent stability. Correspondingly, during the cooling and adsorption periods of the cyclic process, the recombination reactions of the radical species may occur, causing the sorbent to “recover” some stability. These reasons together lead to delayed deactivation in the cyclic processes. Furthermore, in industrial-scale adsorption–desorption units, the adsorption, desorption, and cooling time are much shorter than the conditions used in this cyclic study, leading to even slower sorbent deactivation. In the Supporting Information, we provide various simple mathematical fits based on the cyclic deactivation data to estimate the sorbent lifetime. However, high-fidelity predictions require a much larger data set than is available here.

Like the continuous deactivation studies, we compare and contrast our results from the cyclic experiments with those of the literature. To the best of our knowledge, there have not been any adsorption–desorption cyclic studies on the impact of the copresence of CO_2_ and O_2_ in amine sorbent degradation under dry and humid conditions. The closest relevant example is the study by Heydari-Gorji and Sayari, which performed cyclic studies under humid and dry pure CO_2_ (adsorption at 75 °C and desorption at 105 and 120 °C under N_2_ for 30 min each) and humid 15% CO_2_/N_2_ (adsorption at 50 °C and desorption at 85 °C under humid N_2_ for 30 min each) for 66 cycles. Their results showed a significantly higher loss in CO_2_ uptake under the dry conditions compared to that under the humid conditions studied (dry: 40 and 52% loss, humid: 2 and 3.5% loss, at 105 and 120 °C desorption conditions, respectively).^10^ Similar to the pure humid CO_2_ conditions, the sample treated in humid 15% CO_2_/N_2_ also showed relatively little loss after 66 cycles.^10^ The authors attributed the significant loss under dry conditions to urea formation due to the exposure to high CO_2_ concentrations. In the presence of humidity, however, urea formation is suppressed, and sorbent stability is maintained.^10^

To rationalize the discrepancy in the continuous deactivation behavior of the two studies, we consider the major differences between the two studies, including the support type, CO_2_ uptake temperature, and CO_2_ concentration. First, we note that γ-Al_2_O_3_, the mesoporous oxide support used in this study, is often produced by extracting alumina from metakaolin, a dehydroxylated form of the clay mineral kaolinite.^55^ As a result, γ-Al_2_O_3_ often has trace metal impurities in higher concentration than that of the laboratory-synthesized silica supports, as used by Heydari-Gorji and Sayari.^10^ Mesoporous silica SBA-15, often synthesized in the laboratory from Pluronic P-123 and tetraethyl orthosilicate (TEOS), has notably lower transition metal impurities due to the use of TEOS in the synthesis.^56^ Metal impurities are one of the key contributing factors in initiating alkyl radical species formation and accelerating sorbent deactivation.^49^ A recent study from our group reported higher metal impurities (mainly Fe and Ni) in γ-Al_2_O_3_ (similar to γ-Al_2_O_3_ used in this study) compared to that in SBA-15.^15^ The presence of such transition metals in higher concentrations can lead to O_2_ activation, accelerating oxidation reactions, thereby catalyzing the sorbent deactivation process. As such, the higher concentration of the metal impurities in γ-Al_2_O_3_ compared to that in SBA-15 is likely one reason for the differences observed in the loss in CO_2_ uptake between the two studies.

Additional factors that also may contribute to the differences in stability are the conditions used for the CO_2_ uptake measurement during and after the deactivation experiments (specifically CO_2_ concentration and CO_2_ uptake temperature). In the study by Heydari-Gorji and Sayari,^10^ long-term deactivation experiments were performed under different CO_2_/O_2_/N_2_ concentrations (1–20%/10.5–17%/balance N_2_) and temperatures ranging from 50 to 120 °C for 30 h, whereas in this work, deactivation experiments were performed under 0.04% CO_2_-air, 0.04% CO_2_–N_2_, and CO_2_-free air at 70 °C. Our first-principles modeling indicates that the adsorbed CO_2_ significantly decreases the free-energy barrier of the C–N bond cleavage, likely altering the oxidation rate-determining step from the C–N bond cleavage (either through direct cleavage or alkyl hydroperoxide decomposition^50^) to radical propagation that generates the necessary on-site alkyl radicals before cleaving the C–N bonds. In the CO_2_ concentrations explored in the Heydari-Gorji and Sayari study, the interaction of the amines and CO_2_ occurs quickly. This rapid interaction between CO_2_ and the amine sites due to the high concentration of CO_2_ interlocks the amine chains, slowing down radical propagation, likely preventing the carbamic acid-catalyzed C–N bond cleavage from occurring and the additional CO_2_ and O_2_ from fully accessing the polymer domains. Consequently, in the work of Heydari-Gorji and Sayari, the sorbent stability was more effectively maintained under the deactivation conditions explored. This hypothesis is consistent with the studies probing the diffusion of CO_2_ through supported PEI as a function of CO_2_ concentration and time, where concentrated CO_2_ rapidly cross-links the amine chains and limits further CO_2_ and O_2_ penetration into the bulk of the sorbent,^57^ as well as studies that invoke such behavior to rationalize the cases where comparable CO_2_ uptakes were achieved at both air and flue gas CO_2_ concentrations.^58^

## Conclusions

We have investigated the long-term stability of the PEI/Al_2_O_3_ sorbent under various environments (O_2_, CO_2_, and H_2_O) at an intermediate temperature of 70 °C. The thermal analysis results for oxidative and thermal degradation show a similar impact on the sorbent stability until day 7. After day 7, the oxidative degradation condition becomes dominant, showing significant sorbent deactivation compared to the exclusively thermal conditions. In the copresence of CO_2_ and O_2_ (0.04% CO_2_/21% O_2_) in both humid and dry streams, sorbent deactivation is further accelerated, eliminating the induction period observed in the early stages of oxidative degradation experiments.

In situ HATR-IR experiments reveal that the presence of CO_2_, which leads to surface carbamic acid and ammonium carbamate species, contributes to accelerated oxidative degradation and formation of new primary amine, carbonyl, and imine species, which is the evidence for the C–N and C–H bond cleavages due to oxidative degradation. Based on metadynamics calculations, we hypothesize that the carbamic acid species can catalyze the cleavage of the C–N bonds on adjacent amine chains that contain a carbon-based radical, leading to loss of some amine binding sites.

Sorbent stability studies under close-to-realistic cyclic DAC conditions (30 adsorption–desorption cycles) show a gradual loss in stability (dry: 29%, humid: 52%) under 0.04% CO_2_-air. The loss in capacity during the sorption/desorption cycling is significantly less than the degradation under continuous deactivation conditions, as expected.

The main limitations of this study relative to practical DAC deployments include (i) the use of a TGA for cycling experiments, which differs in flow characteristics from practical DAC gas/solid contactor based on monoliths, fibers, or laminates and the (ii) differing cycle times that result from such TGA use, compared to the more practical contactors. Additionally, desorption is done under a gas purge in these studies, instead of through use of vacuum and/or steam flow, which are favored in some large-scale process designs. Finally, the work here and elsewhere^15^ demonstrate that sorbent degradation is impacted by the nature of the supports used, so further extrapolation is not facile to other systems. Nonetheless, performing studies under these close-to realistic conditions (compared to existing literature studies) aids close comparison to industrial scale DAC operations and provides means for the expedited development and implementation of the sorbent materials with significantly improved stability.

## Supporting Information Available

45 wt % PEI/γ-Al_2_O_3_ sorbent thermogravimetric combustion data, IR spectra (3500–1000 cm^–1^) under various environments, changes in C, H, and N content after heating under dry and humid 0.04% CO_2_-air, cyclic H_2_O uptake under humid N_2_ and mathematical fits to cyclic deactivation data, literature values of CO_2_ uptake loss of 55 wt.% PEI (branched, *M*_n_ ∼ 600)-SBA-15 sorbent after 30 h under prehumidified CO_2_/O_2_/N_2_ mixtures, structures of TETA-carbamic acid/TETA complexes interacting at primary amine and secondary amine sites, and effect of CO_2_ concentration on sorbent stability in the copresence of CO_2_ and O_2_ (PDF)
