# Supplementary material for: Contributions of CO2, O2, and H2O to the Oxidative Stability of Solid Amine Direct Air Capture Sorbents at Intermediate Temperature
Source: ACS Appl Mater Interfaces. 2023 Sep 29;15(40):46790–802. doi: 10.1021/acsami.3c08140 (PMC10571043; doi:10.1021/acsami.3c08140)
Supplement: Supplementary file 1 — am3c08140_si_001.pdf [file am3c08140_si_001.pdf]

Supporting Information for:

Contributions of CO_2, O_2 and H_2O to the Oxidative Stability of Solid Amine Direct Air Capture Sorbents at Intermediate Temperature

Yoseph A. Guta,[1] Juliana Carneiro,[1] Sichi Li,[2] Giada Innocenti,[1] Simon Pang,[2] Miles A. Sakwa-Novak,[3] Carsten Sievers,[1]* Christopher W. Jones[1]*

[1]School of Chemical & Biomolecular Engineering, Georgia Institute of Technology, 311 Ferst Dr., Atlanta, GA 30332, United States
[2]Lawrence Livermore National Laboratory, 7000 East Avenue, Livermore, CA 94550, Unites States
[3]Global Thermostat LLC, 10275 E106th Ave, Brighton, CO 80601, Unites States

*Corresponding authors: carsten.sievers@chbe.gatech.edu; cjones@chbe.gatech.edu;

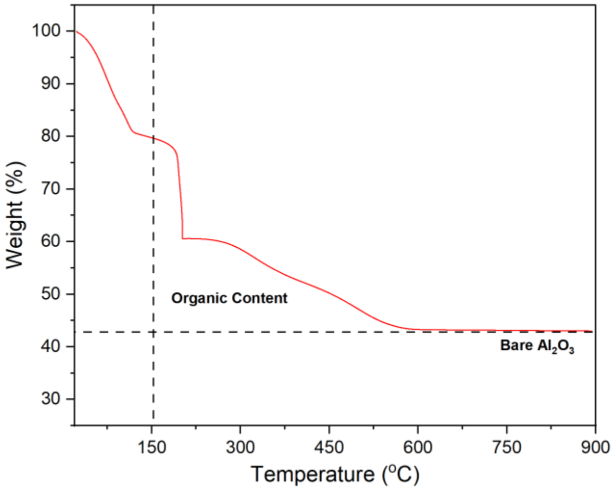

Figure S1. Thermogravimetric combustion of 45 wt.% PEI/Al_2O_3 sorbent measurement showing organic content loss.

Table S1. CO_2 uptake loss of 55 wt% PEI/SBA-15 sorbent after 30 hours under pre-humidified $CO_2/O_2/N_2$ mixtures.[1]

	$CO_2/O_2/N_2$[a]			
Temperature (°C)	1%/17%/82%	5%/14%/81%	7.5%/10.5%/82%	20%/17%/63%
50	n.d.[b]	n.d.	n.d.	0.0
75	n.d.	n.d.	0.1[c]	n.d.
90	n.d.	n.d.	n.d.	1.8
100	70	37	3.0[d]	2.6
120	n.d.	n.d.	n.d.	n.d.

[a]: Streams were humidified, [b]n.d.:not determined, [c]: 120 hours exposure, [d]: 50% uptake loss in the presence of 10.5% O_2/89.5% N_2 stream

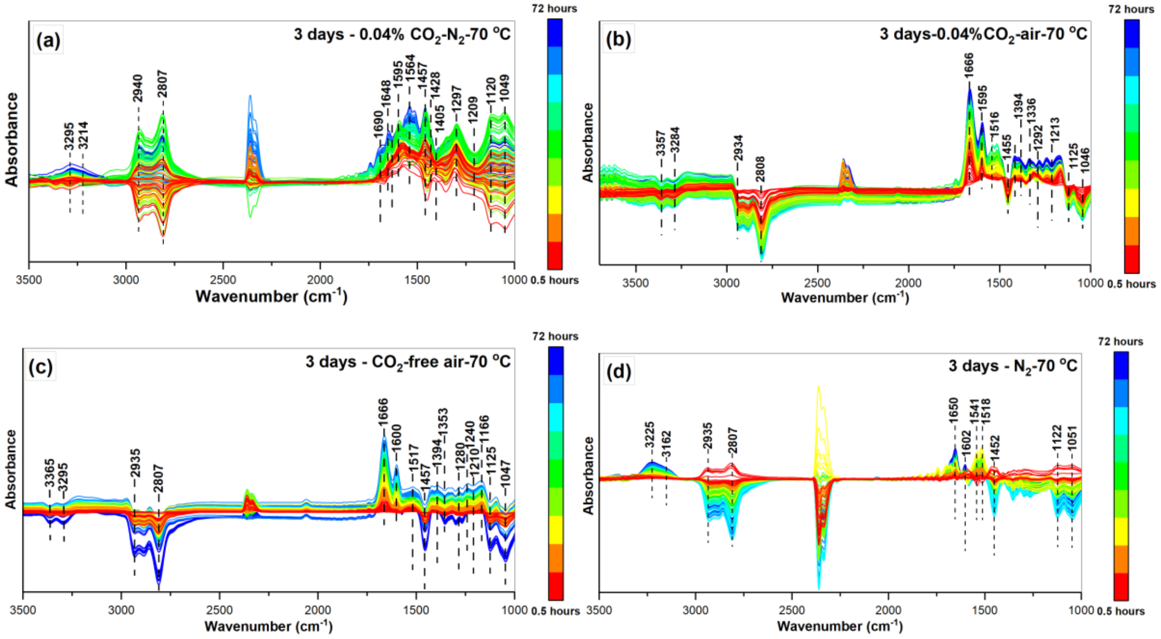

Figure S2. IR spectra (3500 – 1000 cm^{-1}) of PEI/Al_2O_3 deactivation under (a) 0.04% CO_2-N_2, (b) 0.04% CO_2-air, (c) CO_2-free air, and (d) 100% N_2 for 3 days at 70 °C.

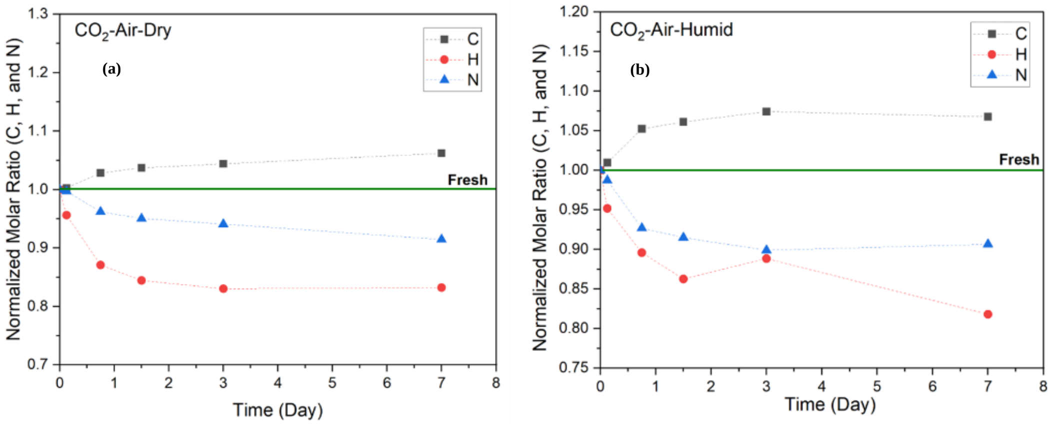

Figure S3. Change in C, H, N content of deactivated 45 wt.% PEI/γ-Al_2O_3 sorbent as a function of time (a) under dry 0.04% CO_2-air (b) under humid 0.04% CO_2-air

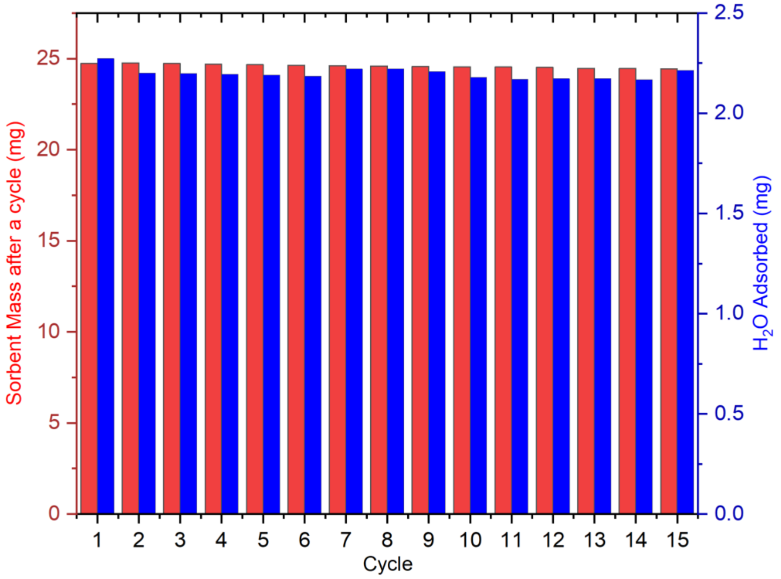

Figure S4. Sorbent mass and H_2O uptake change after 15 cycles under humid N_2.

$$q_{\delta,N} = q_{\delta,0}e^{-kt_{cycle}}$$

Equation S1. $q_{\delta,N}$ is the CO_2 capacity at cycle t_{cycle} (mmol/g sorbent), $q_{\delta,0}$ is the initial CO_2 capacity (mmol/g sorbent), k is the exponential decay constant (cycle^{-1}), and t_{cycle} is the cycle number. (suggested by Azarabadi and Lackner)[2]

Mathematical Fits to Cyclic Deactivation Data

Based on a study by Holmes et al. which assesses adsorbent material performance in industrial-scale DAC operations by modeling sorbent material decay over multiple cycles, the PEI/Al_2O_3 sorbent lifetime beyond the 30 cycles is predicted (**Equation S1**).[2, 3] Accordingly, the CO_2 uptake curves obtained over the 30 cycles of the dry and humid conditions were fitted to an exponential decay model with R-square values of 0.939 for dry and 0.905 for humid. As shown in **Figures S5**, the model predicts the dry cyclic curve to gradually approach complete deactivation after 500 cycles while the humid cyclic curve reaches complete deactivation after about 200 cycles, though the fit in this case is lower. This result indicates that even though the loss in capacity can be similar at shorter cycles between humid and dry conditions, the stability can vary substantially after multiple cycles. Furthermore, the result suggests that even though the presence of humidity prevents urea formation it can enhance oxidative degradation and substantially minimize stability over multiple cycles.

Since the exponential decay model fits are ideal, the CO_2 uptake curves were also fitted to logarithmic and power functions. For both humid and dry cyclic CO_2 uptake curve, neither the logarithmic or power law models fit the data as well as the exponential model. The results for the exponential, logarithmic and power functions are shown in **Figure S5.** From these analyses it is clear that longer term cycling data are needed to provide accurate predictions of sorbent lifetime.

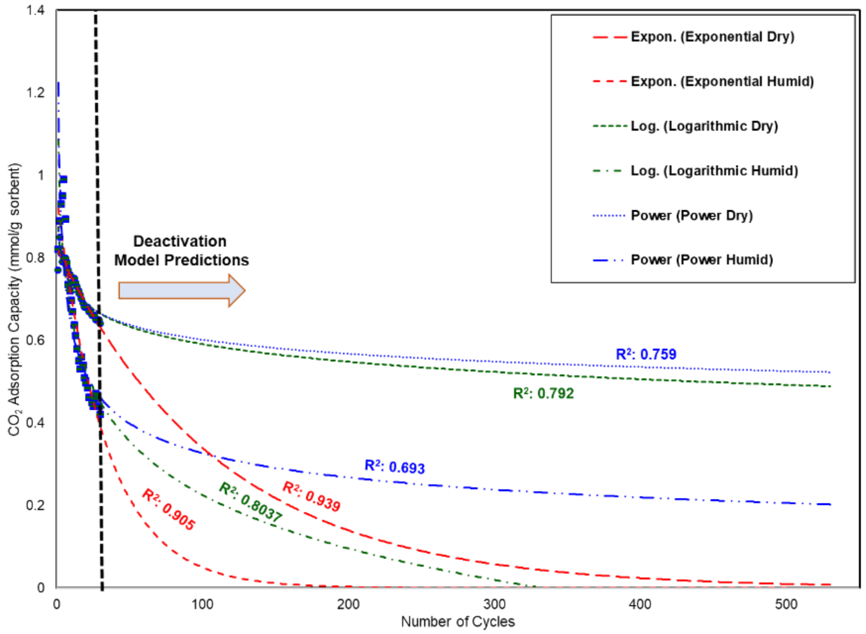

Figure S5. Sorbent lifetime prediction beyond 30 cycles for dry and humid cyclic conditions.

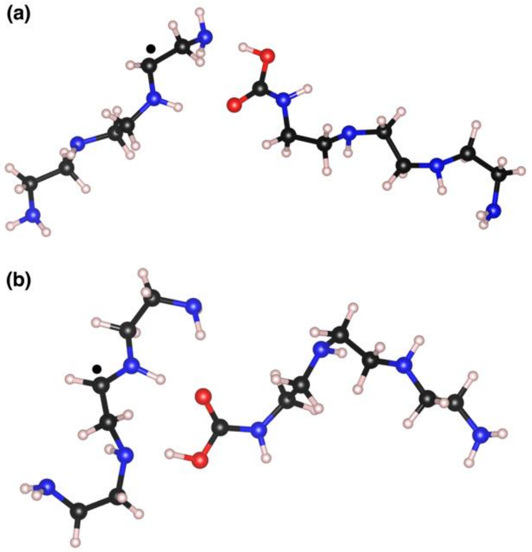

Figure S6. Structures of TETA-carbamic acid/TETA complexes interacting at (a) primary amine and (b) secondary amine sites equilibrated with 10ps of AIMD simulations. Atom color code: C–black, N–blue, O–red, H–pink.

Effect of CO_2 concentration on sorbent stability – in the co-presence of CO_2 and O_2

To confirm the effect of CO_2 concentration in sorbent stability during the co-presence of CO_2 and O_2, deactivation experiments were performed under 1% CO_2-air mixture at 70 °C for 3 hours and 18 hours. **Figure S7 (a and b)** shows a significant increase in sorbent mass under 1% CO_2-air mixture for both 3 hours and 18 hours studies compared to 0.04% CO_2-air mixture where the sorbent mass slightly changes. This increase in sorbent mass under 1% CO_2-air mixture suggests CO_2 adsorption occurring instead of deactivation. **Figure S7c** further supports this claim as the is no

deactivation observed after 18 hours of exposure. 1% CO_2-air mixture is a similar to the gas mixture used in the study by Heydari-Gorji et al. (1% CO_2/17% O_2 balance N_2) and the results obtained below are consistent with their result.

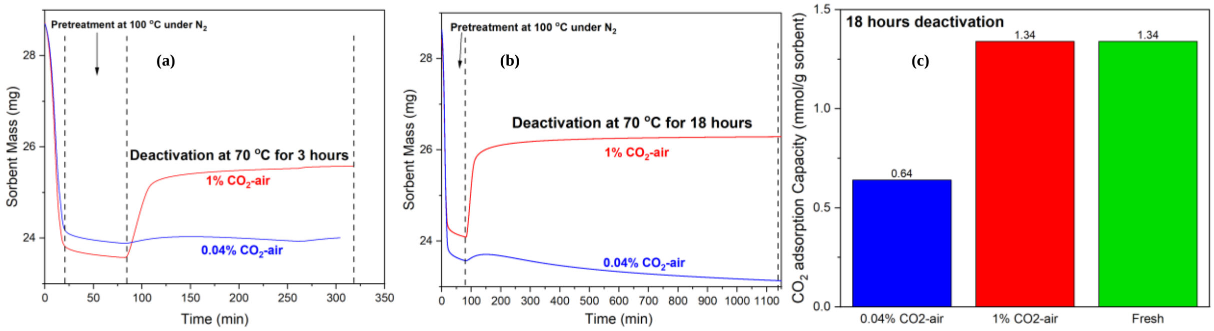

Figure S7. Deactivation profile of 45 wt.% PEI/Al_2O_3 sorbent under 1% CO_2-air and 0.04% CO_2-air mixtures after (a) 3 hours, (b) 18 hours and (c) CO_2 adsorption capacity after 18 hours deactivation under 0.04% CO_2-air and 1% CO_2-air mixtures.

These results support our hypothesis that in the co-presence of CO_2 and O_2, where the CO_2 concentration is above 1%, CO_2 reacts rapidly with amine sites, interlocking the amine chains, slowing down radical propagation, and limiting carbamic acid catalyzed C-N bond cleavage by limiting additional CO_2 and O_2 from fully accessing the polymer domains. However, when the CO_2 concentration is lower, the slower kinetics of CO_2 uptake allow deeper penetration of O_2 and concomitant reactive radical formation.

References

(1) Heydari-Gorji, A.; Sayari, A. Thermal, Oxidative, and CO2-Induced Degradation of Supported Polyethylenimine Adsorbents. *Ind Eng Chem Res* **2012**, *51* (19), 6887-6894. DOI: 10.1021/ie3003446.

(2) Azarabadi, H.; Lackner, K. S. A Sorbent-Focused Techno-Economic Analysis of Direct Air Capture. *Appl Energy* **2019**, *250*, 959-975. DOI: 10.1016/j.apenergy.2019.04.012.

(3) Holmes, H. E.; Lively, R. P.; Realff, M. J. Defining Targets for Adsorbent Material Performance to Enable Viable BECCS Processes. *JACS Au* **2021**, *1* (6), 795-806. DOI: 10.1021/jacsau.0c00127.
